# Hydrogel System with Independent Tailoring of Mechanics, CT, and US Contrasts for Affordable Medical Phantoms

**DOI:** 10.1021/acsmaterialslett.4c01660

**Published:** 2024-09-26

**Authors:** Haoyi Qiu, Jakob Nazarenus, Bernhard Egeler, Tom Thode, Firdaws Osman, Daniar Osmonov, Jörg Bahr, Sören Kaps, Frank-Andre Siebert, Reinhard Koch, Ulf Lützen, Rainer Adelung, Leonard Siebert

**Affiliations:** † Functional Nanomaterials, Department of Materials Science, Kiel University, 24143 Kiel, Germany; ‡ Multimedia Information Processing, Institute for Computer Science, Kiel University, 24118 Kiel, Germany; § Department of Nuclear Medicine, University Hospital Schleswig-Holstein, Campus Kiel, 24105 Kiel, Germany; ∥ Department of Urology, University Hospital Schleswig-Holstein, Campus Lübeck, 23538 Lübeck, Germany; ⊥ Department of Radiation Oncology, University Hospital Schleswig-Holstein, Campus Kiel, 24105 Kiel, Germany; # Kiel Nano, Surface and Interface Science (KiNSIS), Kiel University, 24118 Kiel, Germany

## Abstract

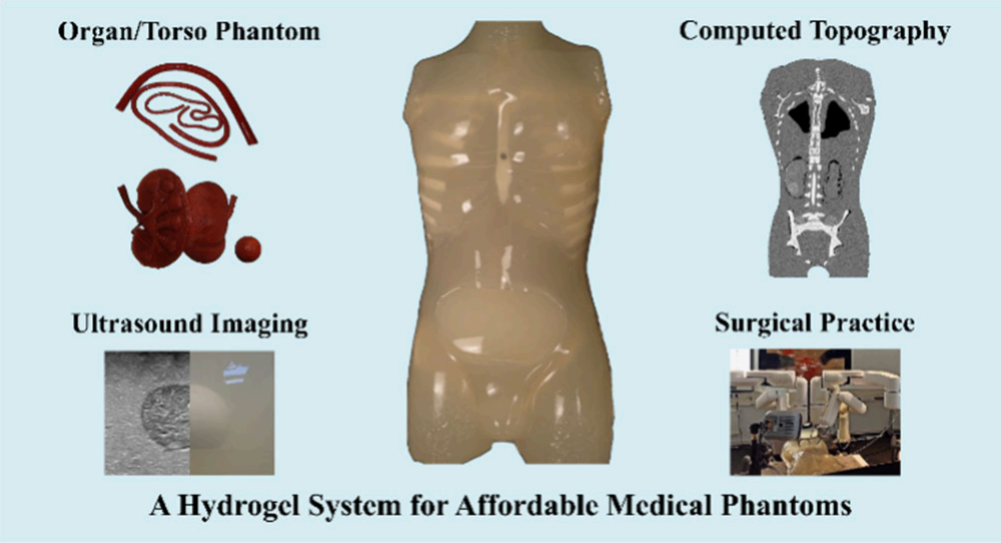

Medical phantoms mimic aspects of procedures like computed tomography (CT), ultrasound (US) imaging, and surgical practices. However, the materials for current commercial phantoms are expensive and the fabrication with these is complex and lacks versatility. Therefore, existing material solutions are not suitable for creating patient-specific phantoms. We present a novel and cost-effective material system (utilizing ubiquitous sodium alginate hydrogel and coconut fat) with independently and accurately tailorable CT, US, and mechanical properties. By varying the concentration of alginate, cross-linker, and coconut fat, the radiological parameters and the elastic modulus were adjusted independently in a wide range. The independence was demonstrated by creating phantoms with features hidden in US, while visible in CT imaging and vice versa. This system is particularly beneficial in resource-scarce areas since the materials are cheap (<$ 1 USD/kg) and easy to obtain, offering realistic and versatile phantoms to practice surgeries and ultimately enhance patient care.

Phantoms are artificial models simulating tissues, body parts, or whole bodies in both medical imaging and surgery practice.^1^ These phantoms help radiologists, medical physicists, and surgeons to practice procedures such as computed tomography (CT), ultrasound (US) imaging, and complex surgical interventions in so-called preprocedure surgical planning.^2,3^ A preprocedural practice provides a significant impact on the positive outcome of complex surgeries like tumor resections.^4−6^ Naturally, a hospital already has both the expertise and the equipment for preprocedural planning and lacks a suitable methodology for creating its own phantoms. Proper preprocedural planning, however, requires the phantoms to be both patient-specific and radiologically, anatomically, and mechanically accurate, which is very challenging.

Single-purpose phantoms are commonly used in medicine, typically designed for device calibration, sing-model imaging or general surgery practice.^7^ The so-called multimodal or multipurpose phantom allows for more than one purpose, i.e., combining multiple imaging modalities and/or surgery practice into one phantom. Commercial phantoms are made of urethane rubber, Zerdine, and epoxy resin and in literature materials like poly(vinyl alcohol), polyvinyl chloride, and silicone are described.^8−17^ Table S1 in the Supporting Information summarizes the details of the commercially available and literature-reported multipurpose phantoms, and none of them is suited for patient-specific care.

Either the described phantom materials are too expensive (i.e., silicone), they contain harmful chemicals (i.e., epoxides), or the fabrication procedure of a realistic phantom is too involved or complex for a hospital setting (i.e., multiple freeze and thaw cycles). Thus, these systems are not ideal for point-of-care fabrication. On top, achieving realistic radiological properties is impossible because CT contrast, US contrast, and the mechanical properties depend on the same material qualities in these systems (e.g., concentration) and can therefore not be varied independently. With these considerations, applying patient-specific radiological phantoms for preprocedural planning is a challenge.^11^

In this study, an economical and food-grade hydrogel system based on coconut fat and ubiquitous sodium alginate has been developed for fabricating multimodal (CT and US) imaging phantoms and subsequent surgical practice. The elastic modulus, CT attenuation, and shear wave velocity of the composite can be independently tailored, even beyond the physiologically reasonable ranges. The cost-effectiveness, the harmlessness of the ingredients, and the simplicity of the fabrication enable patient-specific, radiologic preprocedural surgical phantoms for the first time.

Alginate is a polysaccharide that is derived from marine brown algae.^25^ The chemical structure of sodium alginate is shown in Figure 1A, composed of repeating units of two different monosaccharides, α-l-guluronic acid and β-d-mannuronic acid. These monosaccharides are linked together by glycosidic bonds to form a long chain-like structure. As demonstrated in Figure 1A, sodium alginate as a water-soluble polymer can be ionically cross-linked by the addition of divalent cations (e.g., Ca^2+^). No significant influence on the cross-linking process can be observed when adding fat. However, the CT attenuation becomes lower due to the decreased density upon increasing the fat content (Figure 1B). In order to see the distribution of coconut fat in the material system, Raman spectroscopy was conducted. As shown in Raman images (Figure 1D), the size of the fat inclusions in the alginate is below 20 μm, therefore each pixel of the CT scan is the average of the X-ray attenuation of alginate, fat and water. The shear wave velocity in US imaging is linked to the elastic modulus and the density of the materials. The elastic modulus, therefore, the shear wave velocity can be increased by increasing the cross-linking with Ca^2+^ ions. This can be seen in Figure 1C, where a layer of lower cross-linking degree (0.2 wt % CaCO_3_) is sandwiched between more strongly cross-linked (0.6 wt % CaCO_3_) layers. The layers with the higher cross-linking appear brighter, and their contrast is higher because the shear waves can travel faster in this medium.

**Figure 1:**
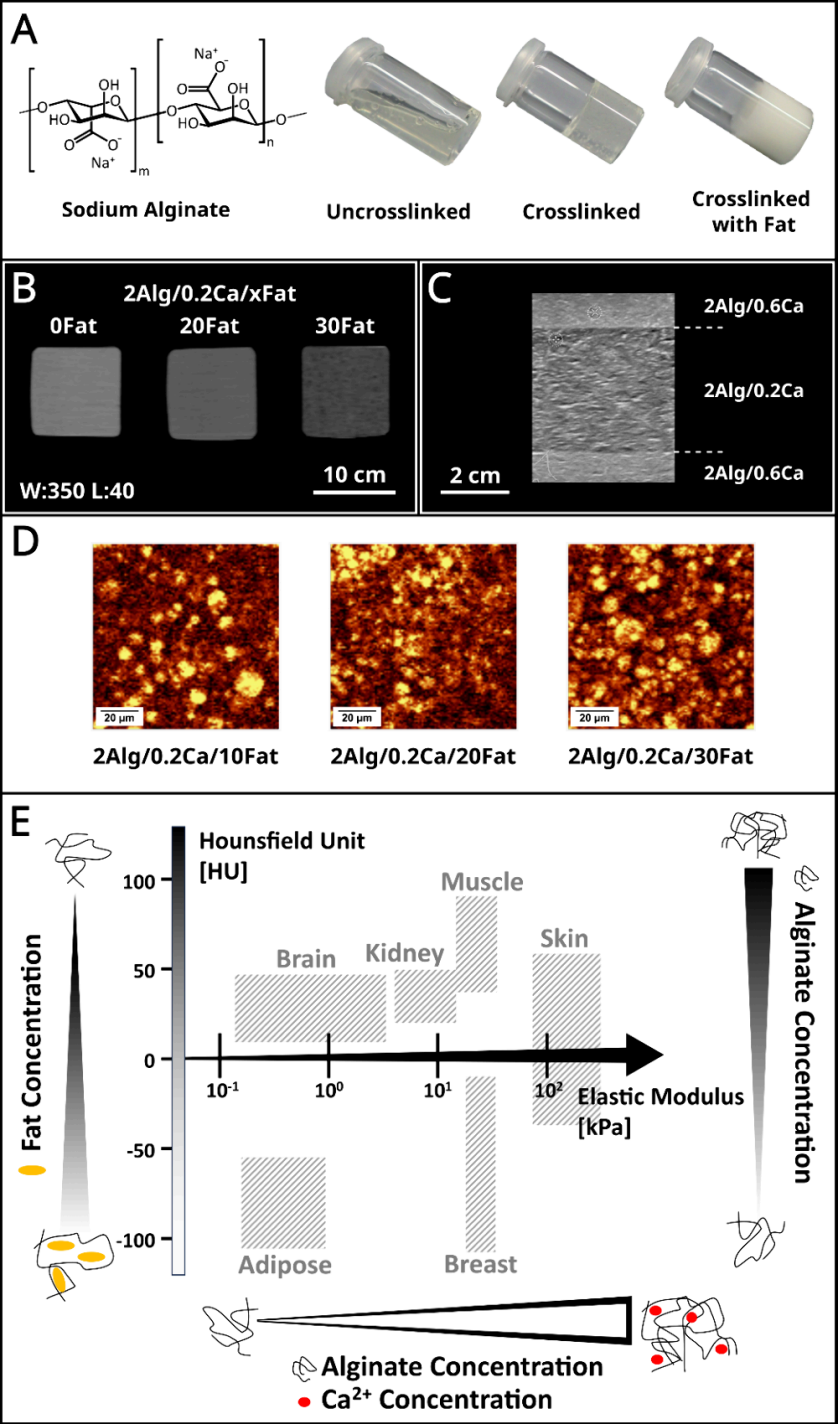
(A) Chemical structure of sodium alginate and photos of un-cross-linked sodium alginate, cross-linked alginate with Ca^2+^, and Ca^2+^ cross-linked alginate with fat addition. (B) CT images of material variations: 2 wt % alginate cross-linked with 0.2 wt % CaCO_3_ and with different coconut fat additions (0, 20, and 30 wt %). The window level and window width are set at 40 and 350, respectively. The increase in fat concentration leads to a lower contrast, i.e., lower CT attenuation. The numbers in front of the ingredients in the figures represent weight percentages (e.g., 2Alg/0.2Ca/10Fat represents 2 wt % alginate/0.2 wt % CaCO_3_/10 wt % fat). (C) US images of material variations: 2 wt % alginate with different cross-linking degrees (0.2, 0.4, and 0.6 wt %) prepared in layers. The difference in shear wave velocity can be seen by the lower contrast of the less cross-linked middle layer in relation to the higher cross-linked upper and lower layers. (D) Filtered Raman images of material variation: 2 wt % alginate cross-linked with 0.2 wt % CaCO_3_ and with different coconut fat additions (10, 20, and 30 wt %). The fat signal appears bright yellow with respect to the bending CH_2_ vibration at 1445 cm^–1^. (E) Demonstration of how the Hounsfield unit and elastic modulus of the hydrogel composites developed in this study can be adjusted independently to achieve the required values of human tissue and organs. The Hounsfield unit and elastic modulus of human tissue and organs were obtained from the previous studies.^18−24^

Figure 1E demonstrated that by changing the concentration and cross-linking degree of alginate and the amount of coconut fat addition, the developed system can simulate the properties of specific tissues and organs. With increasing the cross-linking, stiffer tissues like skin can be simulated, while with higher fat content more fatty tissues like adipose tissues and breasts can be simulated. A low-fat content and a high alginate concentration with high cross-linking results in tough tissues with high HU values like muscles.

It is difficult to obtain any combination of HU, elastic modulus or shear wave velocity if they cannot be tuned independently.^11^ In Figure 2A, the HU, elastic modulus, and their corresponding standard deviation of different material variations are shown. It demonstrates how the individual properties can be tweaked without altering other properties significantly (grouped in red and yellow circles), and a wide range (value of the real organs or tissues and even beyond) of elastic modulus and CT attenuation can be achieved. By varying the concentration of alginate, cross-linker, and coconut fat (2–15 wt %, 0.1–10 wt %, and 1–30 wt %, respectively), the HU and elastic modulus can be adjusted independently in a range of around −120–120 HU and 1–250 kPa, respectively. The details about how the individual parameters affect the properties are shown in Figure 2B–E and the material composition of the data points in Figure 2A can be found in Figure S10. Figure 2B shows the influence of the cross-linking degree on the elastic modulus and CT attenuation. An increase in the concentration of cross-linker (Ca^2+^) results in a significant increase in the elastic modulus, while having no significant effect on the CT attenuation in Hounsfield Unit. The addition of coconut fat is observed to significantly decrease the CT attenuation in the Hounsfield Unit, as shown in Figure 2C. However, the elastic modulus is not affected by the addition of coconut fat. The results presented in Figure 2D and 2E indicate that it is possible to independently manipulate the CT attenuation and shear wave velocity through the addition of coconut fat and alteration of the cross-linking degree, respectively.

**Figure 2:**
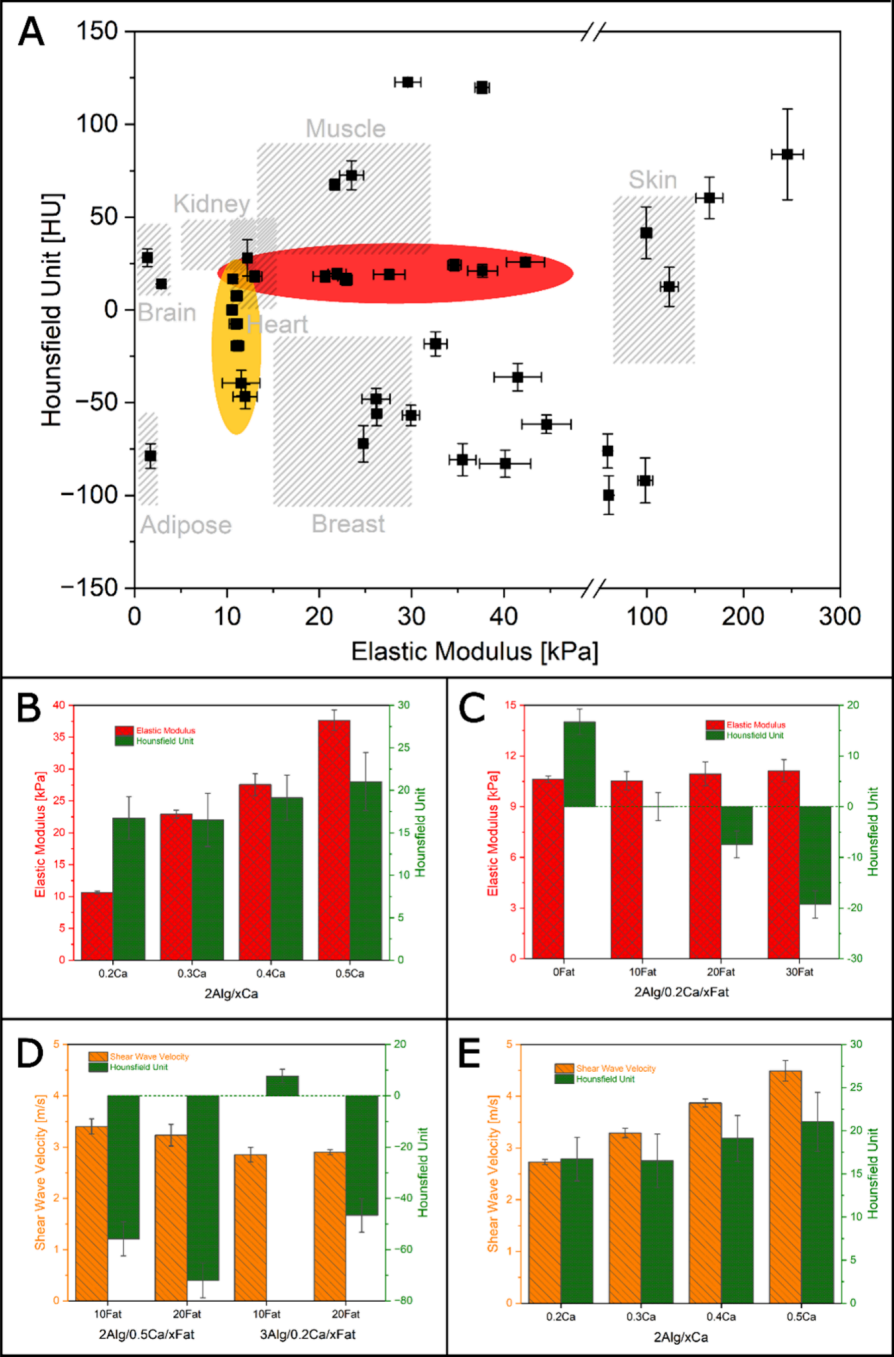
(A) The HU, elastic modulus, and their corresponding standard deviation of different material variations, showing the influence of concentration and cross-linking degree of alginate and fat addition on the elastic modulus and CT attenuation (HU). An increase in the concentration of cross-linker (Ca^2+^) results in a significant increase in the elastic modulus, while having no significant effect on the CT attenuation in HU (grouped in red circle). The addition of coconut fat significantly decreases the CT attenuation in HU while the elastic modulus is not affected (grouped in the yellow circle). A wide range of elastic modulus and CT attenuation can be achieved even beyond the real organs or tissues by adjusting the concentration of alginate, degree of cross-linking, and the addition of coconut fat. The Hounsfield unit and elastic modulus of human tissue and organs were obtained from the previous studies.^18−24^ (B) Influence of cross-linking degree and (C) fat addition on the elastic modulus and CT attenuation. (D,E) Influence of alginate concentration, cross-linking degree, and fat addition on the CT attenuation and shear wave velocity of material variations.

One of the main points for the actual fabrication of patient-specific phantoms is the simplicity and cost-effectiveness of the production process. A minimum of expertise should be required to fabricate the phantoms so that any setting, especially a hospital setting, is sufficiently equipped for their production. The production of this hydrogel system is simple and cost-effective. After all of the harmless ingredients were mixed, the liquid hydrogel solution can be cast into different and suitable sizes and shapes for a specific purpose. As shown in Figure 3, alginate solutions with different amounts of calcium ion and fat addition were cast into shapes of letters T and F. The letters of the same variation were placed diagonally inside a box filled with a third material variation. The CT and US measurements further confirmed that the CT attenuation and shear wave velocity can be adjusted independently. The letters (upper left T and lower right F) having the same cross-linking degree and different fat addition as the matrix variation are visible in the CT image due to the density difference to the matrix but invisible in the US image due to the similar elastic modulus. On the other hand, the letters (lower left T and upper right F) having the same fat addition but different cross-linking degrees as the matrix variation are only visible in the US image due to their different elastic modulus to the matrix. They are invisible in the CT image since the density of the variation is not significantly influenced by the cross-linking degree.

**Figure 3:**
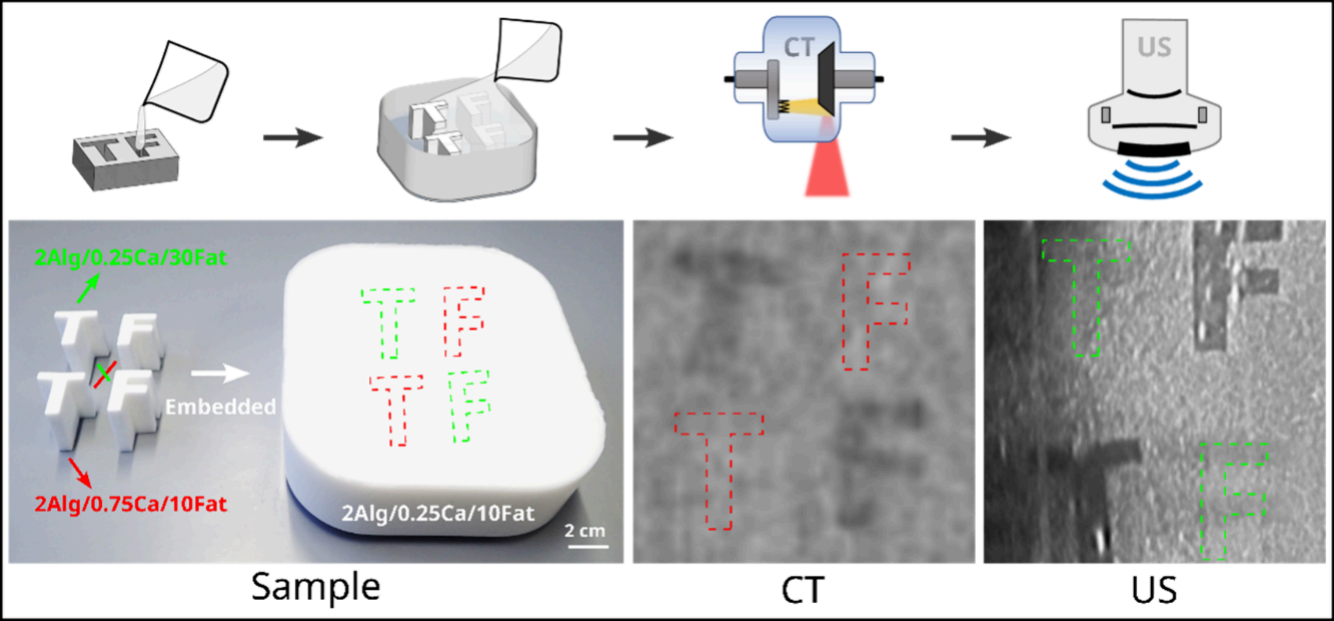
Samples of 2 wt % alginate with different fat addition and cross-linking degrees (10 and 30 wt % fat, 0.25 and 0.75 wt % CaCO_3_) cast in the shape of letters T and F, embedded in a matrix variation (2 wt % alginate/0.25 wt % CaCO_3_/10 wt % fat). The CT and US images of the matrix contain the letters T and F. The window level and window width of the CT image are set at 40 and 350, respectively.

In that way, tailored hydrogel constructs can be molded into arbitrary shapes, with each construct having its own distinct set of properties, mimicking real organs and tissues. The photograph presented in Figure 4A depicts blood vessel, kidney, and tumor phantoms that were fabricated by casting the composite material into 3D-printed molds. The blood vessel, kidney, and tumor phantoms were incorporated into an acrylic torso shell. One of the kidney phantoms contains an embedded spherical tumor phantom. The torso phantom was fabricated by casting the composite material into the prepared torso shell, as illustrated in Figure 4B. The resulting CT image of the torso phantom, shown in Figure 4C, highlights the presence of the tumor inside the right kidney as a result of the varying CT attenuation.

**Figure 4:**
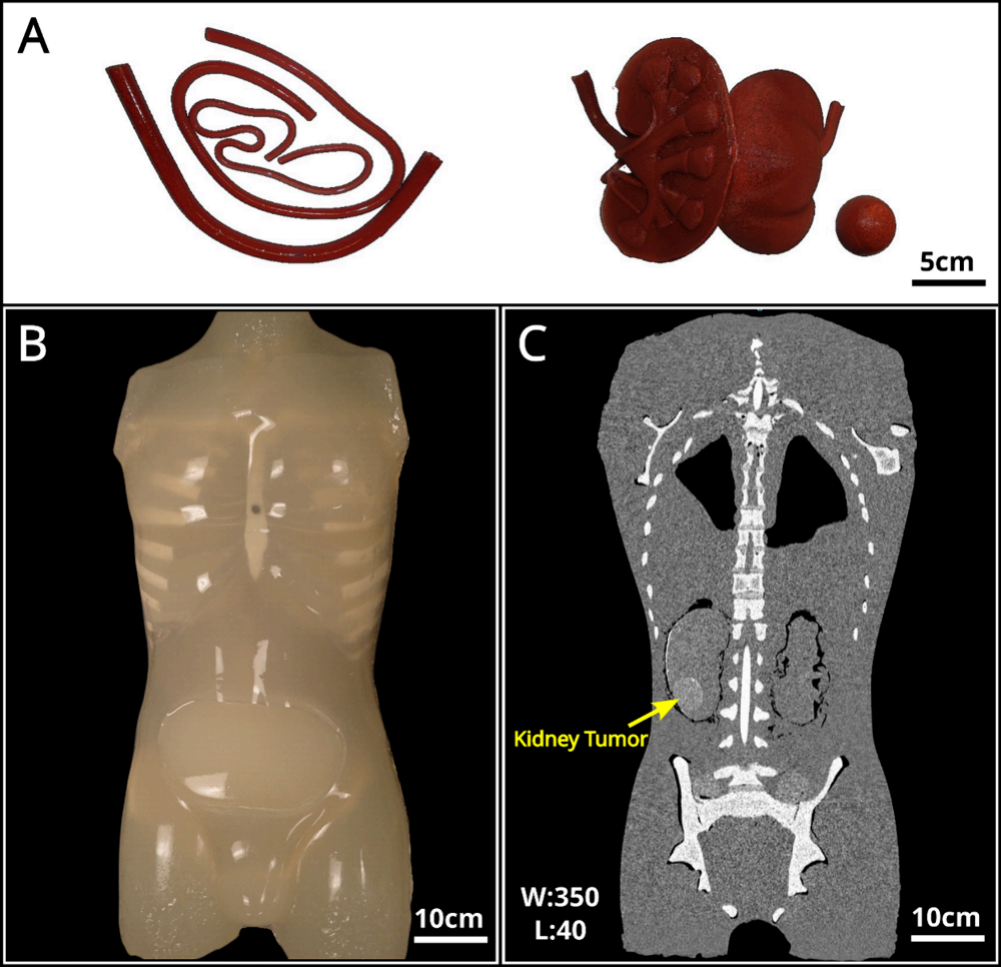
(A) Photograph of the blood vessel, kidney, and tumor phantoms prepared by casting the composite material into 3D-printed molds. (B) Photograph of the torso phantom fabricated by casting. (C) CT image of the phantom with artificial kidney and tumor. The window level and window width are set at 40 and 350, respectively.

Single-purpose phantoms have been previously developed to simulate either CT or US imaging or for mechanical surgery practice.^7^ However, CT, US, and mechanical properties could so far not be tailored independently for multipurpose applications.^26,27^ These phantoms are typically expensive to manufacture and nonbiodegradable.^28^ Thus, they are not suitable for use in destructive surgery practice, especially in patient-specific surgery training.^29^ Additionally, the cost of a one-time patient-specific phantom for surgical planning and practice should be inexpensive. In the present study, a cost-effective hydrogel composite based on alginate and coconut fat is developed for fabricating multipurpose medical phantoms. The CT, US, and mechanical properties can be tailored independently to meet all the requirements of different organs and tissues, making it suitable for creating anatomically accurate whole-body phantoms. Whole 40-kg torso phantoms can be fabricated for material costs below $ 50 USD, in comparison to commercial single-purpose phantoms that sell for over $ 10,000 USD (For more detailed cost analysis, refer to the Supporting Information). Due to the easy fabrication process, students or doctors can even quickly create phantoms themselves and receive timely training.

The capability of the materials to fulfill their purpose in a phantom is the greatest requirement for a patient-specific approach. For this, recipes for the materials system are necessary that can express any of the desired quantities. The demonstrated approach using alginate and fat exhibits these qualities. The elastic modulus of the composite can be tuned by changing the cross-linking degree of the alginate by varying the concentration of divalent cations (Ca^2+^) in the alginate solution. When alginate is cross-linked, the carboxylate groups on the alginate chains form a three-dimensional network that holds the material together. When the cross-linking degree of alginate is increased, the number of cross-links between the alginate chains increases, and the material becomes stronger and stiffer. The stiffness of the chains, however, does not affect the CT attenuation, and therefore, the HU is independent of the cross-linking degree. The exchange of Na^+^ to Ca^2+^ in the phantom does not influence the CT attenuation.

However, due to the fact of ionic cross-linking, the tear resistance of alginate is low. Therefore, for real surgical practice, the tear resistance might not be sufficient for stronger handling, such as the sewing of phantom skin. One approach to improve the tear resistance of alginate is to incorporate natural fibers, such as cellulose, into it.^30^

The CT attenuation of a material depends on the density of the material^31,32^ and can therefore be reduced by the addition of (coconut) fat. Since coconut fat has a lower density than the alginate mixture (mostly water), the phantom with higher fat addition absorbs fewer X-rays and therefore decreases the overall CT attenuation of the mixture. Since the fat sits between the cross-linked chains of alginate, which determine the stiffness (for details see Figure 1D and Supporting Information Figure S13) its addition does not significantly impact the elastic modulus of the material. Therefore, elastic modulus and CT attenuation can be tailored independently of each other, as can be seen in Figure 2.

Shear wave velocity in US imaging refers to the speed at which shear waves propagate through a material, and is directly proportional to the elasticity (or stiffness) of a material.^33^ It can be adjusted by changing the cross-linking degree of alginate since cross-linking affects the elastic modulus of the material. However, fat addition decreases the density, and therefore, this change has to be compensated for by increasing the alginate concentration. With the ability to independently tailor the US and CT properties, the developed material system has a clear advantage compared to the previously reported materials. For example, Hungr et al. have used PVC mixtures in different elasticities to prepare the prostate phantom by varying the ratios of hardener to softener.^11^ However, when tailoring the US contrast by changing the mixing ratios, the density, therefore the HU is also significantly influenced.

In summary, the present study demonstrates a new and easy approach to fabricating medical phantoms using alginate/coconut fat-based hydrogel composites. The elastic modulus, CT attenuation, and shear wave velocity of the composite can be tailored independently by controlling the concentration of alginate, the cross-linking degree of alginate, and the amount of coconut fat, enabling the simulation of specific tissue properties and thereby create anatomically accurate and cost-effective whole-body phantoms. The developed approach can ultimately lead to better patient care, especially in resource-limited and developing regions, as the phantoms can be produced on a large scale and provide better training in diagnostic imaging and surgical practice.

## Experimental Section

### Materials

All involved materials are harmless, and most of them are even food-grade and common ingredients in food preparation. Food-grade sodium alginate and the plant-based surfactant decyl glucoside were purchased from Dragonspice Naturwaren (Reutlingen, Germany). The food additive glucono-deltalactone (GDL) was supplied by Hausschlachtebedarf (Sangerhausen, Germany). The calcium carbonate (CaCO_3_) was purchased from Sigma-Aldrich (Taufkirchen, Germany). The food-grade coconut fat was produced by Peter Kölln GmbH & Co. KGaA (Elmshorn, Germany). All the ingredients are cheap, have high accessibility and can be readily supplied by local food suppliers.

### Phantom Preparation

The sodium alginate and CaCO_3_ powders were first dispersed in distilled water for 24 h. The surfactant decyl glucoside and coconut fat were mixed into the alginate dispersion and the mixture was degassed for about 5 min to remove any air bubbles. The GDL powder was dissolved in the distilled water and poured into the mixture immediately. The final mixture was stirred slightly by hand and cast into the desired shape.

Material variations with different concentrations and cross-linking degrees of alginate and different coconut fat addition were prepared. For example, 2 wt % alginate cross-linked with 0.2 wt % CaCO_3_ and with 20 wt % coconut fat addition was prepared following the steps: 2 g of sodium alginate was mixed into 48 g of distilled water at room temperature, hereafter named premixture 1. For homogeneous mixing, the premixture 1 was stored at room temperature for 24 h before the next step. 0.2 g of CaCO_3_ microparticles were mixed into 24.8 g of distilled water for 1 min to obtain a homogeneous dispersion (premixture 2). Premixture 2 was added to premixture 1 and stirred for 2 min until the dispersion is homogeneous, named premixture 3. 1.25 g of surfactant decyl glucoside was added to premixture 3 and stirred for 2 min until fully mixed, named premixture 4. 23.75 g of molten coconut fat (molten at 85 °C for 1 h) was added to premixture 4 and stirred vigorously for 5 min until it was homogeneous, named premixture 5. Premixture 5 was degassed in a vacuum chamber for 10 min to remove air bubbles. 0.71 g of GDL was added into 24.29 g of water and stirred for 15 s until it was completely dissolved, named premixture 6. Immediately after preparing premixture 6, it was poured into the degassed premixture 5 and stirred for 30 s gently to not introduce air bubbles to obtain the final mixture. The final mixture was poured into 3D-printed polylactic acid molds (shown in Figure S1) and cast into the desired shape. The details for samples and phantom preparation are illustrated in the Supporting Information.

### Compression Test

To evaluate the mechanical properties of the material variations, compression tests were conducted with the universal testing machine (quickTest Prüfpartner GmbH, Langenfeld, Germany, no longer existing) according to the standard DIN EN ISO 3386. The cylindrical-shaped compression test specimens (10 mm in diameter and 10 mm in height) were prepared by casting the mixture into the 3D-printed polylactic acid (PLA) molds (for details, see Supporting Information). The compression tests were conducted at room temperature with a compression speed of 10 mm/min. Seven specimens were measured for each material variation, from which the mean elastic modulus with the corresponding standard deviation was calculated. The elastic modulus was obtained according to Hooke’s law in the elastic region between 1% and 10% strain.

### Computed Tomography

To evaluate the CT attenuation (determined by density) of the material variations, the material samples (∼100 mm × 90 mm × 50 mm) were measured by a Symbia Intevo 6 SPECT/CT Hybridscanner (Siemens AG, Munich, Germany). The slice thickness was set to 1.25 mm, and the convolution kernel was B08s. The acquired image data sets were archived in a DICOM format. The CT attenuation was represented by the Hounsfield unit (HU). The mean HU of the region of interest (sphere with a radius of 30 mm) was calculated from the DICOM files by a self-developed Python-based software (for details see Supporting Information). The rescale intercept and rescale slope were −1024 and 1, respectively. To better visualize different material variations in the CT images, the proper window center and width were selected and noted in the corresponding images.

### Sonography

The US images were recorded using B-Mode Imaging by a Philips Ultrasound System EPIQ Elite (Koninklijke Philips N.V, Amsterdam, The Netherlands). Additionally, the shear wave velocity of the material variations was measured, where the measurement depth was set to 25 mm. Three areas with confidence higher than 50% were measured, and the mean values were calculated.

### Raman Spectroscopy

The influence of the cross-linking and fat addition on the properties of alginate and fat distributions was investigated by Raman Spectroscopy (Witec Alpha 300RA, Ulm, Germany) equipped with a 532 nm wavelength Ar laser. The Raman images were calculated by filtering the bending CH_2_ vibration at 1445 cm^–1^.

## Supporting Information Available

Summary of current phantoms, cost analysis, sample preparation, compression test, process of CT data, torso phantom preparation, CT measurement, US measurement, robotic surgery, antimold feature of coconut fat, independently tunable material properties, scanning electron microscopic images, torso phantom and surgical practice (PDF)
