# Supplementary material for: Hydrogel System with Independent Tailoring of Mechanics, CT, and US Contrasts for Affordable Medical Phantoms
Source: ACS Mater Lett. 2024 Sep 26;6(10):4847–53. doi: 10.1021/acsmaterialslett.4c01660 (PMC11463687; doi:10.1021/acsmaterialslett.4c01660)
Supplement: Supplementary file 1 — tz4c01660_si_001.pdf [file tz4c01660_si_001.pdf]

Supporting Information

A Hydrogel System with Independent Tailoring Mechanics, CT and US Contrasts for Affordable Medical Phantoms

Haoyi Qiu[1], Jakob Nazarenus[2], Bernhard Egeler[3], Tom Thode[1], Firdaws Osman[1], Daniar Osmonov[4], Jörg Bahr[1], Sören Kaps[1], Frank-Andre Siebert[5], Reinhard Koch[2], Ulf Lützen[3], Rainer Adelung[1,6,], Leonard Siebert[1,6,*]*

[1]Functional Nanomaterials, Department of Materials Science, Kiel University, 24143 Kiel, Germany

[2]Multimedia Information Processing, Institute for Computer Science, Kiel University, 24118 Kiel, Germany

[3]Department of Nuclear Medicine, University Hospital Schleswig-Holstein, Campus Kiel, 24105 Kiel, Germany

[4]Department of Urology, University Hospital Schleswig-Holstein, Campus Lübeck, 23538 Lübeck, Germany

[5]Department of Radiation Oncology, University Hospital Schleswig-Holstein, Campus Kiel, 24105 Kiel, Germany

[6]Kiel Nano, Surface and Interface Science (KiNSIS), Kiel University, 24118 Kiel, Germany

[*]Corresponding author: Rainer Adelung (ra@tf.uni-kiel.de); Leonard Siebert (lesi@tf.uni-kiel.de)

Summary of the current phantoms

Table S1 summarizes the types, materials, features, pros and cons of the commercially available and recently developed multipurpose phantoms.

Table S1: Commercially available and recently developed multipurpose phantoms.

Types		Materials	Features	Pros & Cons
Commercial	Multi-Purpose & Endoscopic Phantom (CIRS Model ATS 570)	Urethane rubber	• US imaging, accommodating various probes including linear, sector, endoscopic and mechanical sector probes	+ Commercially available from CIRS, Norfolk, USA<br>− Relatively high cost<br>− Not for hands-on surgical training

	Multi-Purpose, Multi-Tissue Ultrasound Phantom (Model 040GSE)	Zerdine® (solid elastic hydrogel)	• US imaging, for performance and quality assurance testing	
	Triple Modality 3D Abdominal Phantom (Model 057A)	Z-Skin™ elastomer, epoxy resin, Zerdine®	• Multimodal imaging (US, CT)<br>• Multiple biopsy insertions	
	Multipurpose-Phantom N365	Urethane elastomer, acryl, nylon	• US imaging, from all four side walls	+ Commercially available from Kyoto Kagaku, Kyoto, Japan<br>– Relatively high cost<br>– Not for hands-on surgical training
	Multipurpose Chest Phantom N1 "LUNGMAN"	Polyurethane, epoxy resin	• CT imaging, radiation absorption and HU number very close to the human body	
	Take-Apart Pixy (RS-103)	Unknown	• CT imaging, highly customizable including custom pathology and injury	+ Commercially available from Radiology Support Devices Inc., Long Beach, USA<br>– Relatively high cost<br>– Not for hands-on surgical training
Research Work	Prostate phantom [1]	Polyvinyl chloride	• Multimodal imaging (US, CT)<br>• Needle-insertion procedures	+ Simple manufacturing<br>+ Relatively low materials costs<br>– Non-biodegradable<br>– Small dimension demonstrated<br>+ Not for hands-on surgical practice
	Brain Phantom [2]	Polyvinyl alcohol	• Multimodal imaging (US, CT)<br>• Brain Tumor Surgery Planning	+ Patient-specific<br>+ Biodegradable<br>– Freeze-thaw cycles required, increases preparation time<br>– Moldy over time (Addition of potassium sorbate extends its shelf-life)<br>– Small dimension demonstrated<br>– Uncontrollable mechanical properties<br>– Cannot adjust properties independently
	Breast phantom [3]	Ballistic gelatin, Metamusil™	• Multimodal imaging (US, CT)<br>• Training for minimally invasive image-guided procedures (e.g., catheter or needle insertion)	+ Biodegradable<br>+ Relatively low materials costs<br>– Contrast agent required for CT contrast<br>– Relatively low mechanical properties<br>– Small dimension demonstrated
	3D-printed breast phantom [4]	Polyvinyl chloride	• Multimodal imaging (US, CT)	+ Additive manufacturing, ease of customization<br>+ Relatively low materials costs<br>– Non-biodegradable<br>– Small dimension demonstrated<br>– Not for hands-on surgical practice
	Patient-specific cardiac phantom [5]	Silicone, Polyvinyl alcohol	• Multimodal imaging (US, CT)<br>• Simulate a variety of minimally invasive cardiac procedures	+ Patient-specific<br>– Silicone non-biodegradable<br>– Relatively high materials cost<br>– Small dimension demonstrated
This work		Alginate, coconut fat	• Multimodal imaging (US, CT)<br>• Robotic surgery training<br>• Practicing for patient-specific surgeries	+ High material availability (also for developing countries)<br>+ Very low materials costs<br>+ Simple manufacturing<br>+ Properties adjustable independently and precisely<br>+ Specific value adjustable in a wide range with high-resolution<br>+ Whole body with anatomically correct organs (discrete parts possible)<br>+ Ready to assembly<br>+ Large scale application<br>+ Biodegradable & eco-friendly<br>+ Anti-mold with coconut fat<br>+ No hesitation to cut and do surgery practice<br>+ Fast custom desirable for specific training<br>+ Practice difficult surgeries in time

Cost analysis

One of the crucial reasons, why phantoms are not ubiquitous for patient-specific care is inaccessibility. This quality is reflected in both the price of commercial phantoms, their long shipping times and the difficulty of the production, rendering an in-house phantom fabrication impossible. The cost for commercially available phantoms is usually high. For example, the CT phantom from RSD (RS-107CT, Radiology Support Devices, Inc. United States) costs about $ 43,000 USD while the US training phantom from Erler-Zimmer (R16590, Erler-Zimmer GmbH & Co. KG, Germany) costs about $ 23,000 USD [6]. The cost of a phantom is related to the materials used, the equipment needed for fabrication (such as 3D printers, molds, etc.), the fabrication time and conditions, details and accuracy of the phantom, distribution (including shipping and handling), insurance, etc [7,8]. Among all these factors, the material costs are the first important factor influencing the price, since it will affect the costs of the subsequent steps. When only considering the material costs of commonly used materials for phantom fabrication (summarized in Table S1), polyurethane is around $ 40 USD/kg (Polytek Development Corp., Pennsylvania, USA; Nedform b.v., Geleen, Netherlands). For silicone, the price is around $ 25 USD/kg to $ 50 USD/kg (Silikonfabrik Inh. Alexander Blioch, Bad Schwartau, Germany; Reschimica srl, Barberino Tavarnelle, Italy). In contrast, the material developed in this study is cheaper than $ 1 USD/kg, and the total material cost is less than $ 50 USD/kg for fabricating a 40 kg torso phantom. Moreover, the production time is very relevant for patient-specific care and the current approaches do not completely address this factor. For example, the phantom constructed from PVA-C is very time-consuming due to the 24-hour long freezing cycles [6,9]. The material developed in this study can be crosslinked and solidified within 1 hour. The total fabrication time of a phantom also depends on its details and accuracy. Just to give a rough idea, the 40 kg torso phantom with kidney and tumor presented here can be fabricated within about 3 hours by one person. This feature is highly beneficial to construct patient-specific phantoms in time. Additionally, the materials in this study, i.e., alginate, fat, water, calcium carbonate and GDL are all usually produced in large quantities for the food industry and therefore easily accessible. The costs for labor in other cases can also be higher, since the chemicals (polyurethane [10,11], etc.) involved in phantom fabrication are harmful (requiring more training for the fabrication personnel and more protective equipment (hoods, chemically resistant gloves, etc.). Since all the materials used in this study are food grade, it doesn't require special working conditions or protective equipment which can lower the costs. The

Sample preparation

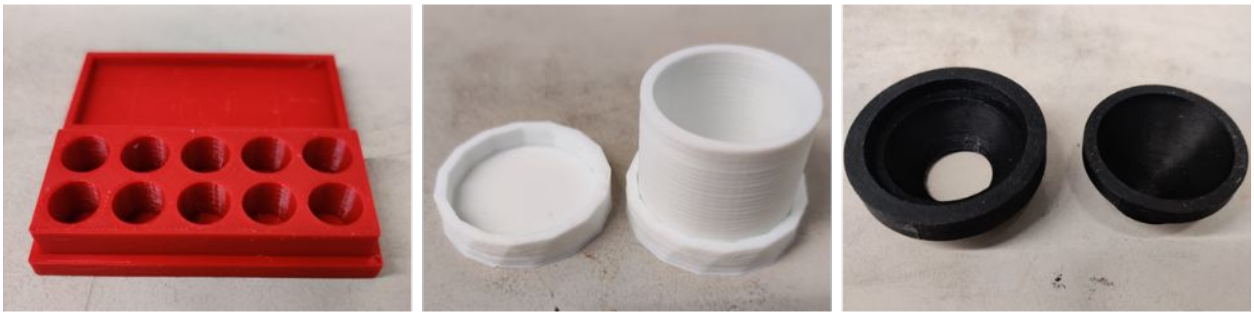

Figure S1: 3D-printed PLA molds for casting the materials into the desired shape.

The crosslinking of the hydrogel system is time-triggered. The dissolution of GDL in water triggers a hydrolysis reaction of GDL to gluconic acid, which is capable of dissolving $CaCO_3$. Two solutions are prepared, one containing the alginate, fat, a dispersion of $CaCO_3$ microparticles and any other desirable additives like coloring, and the second solution containing water and GDL. After mixing these two solutions the hydrolysis and the slow dissolution of $CaCO_3$ give about a 15-minute window for casting, until the mixture starts to set. When casting, care has to be taken to not introduce air bubbles, since they impact both the CT attenuation and the elastic modulus significantly. The $CaCO_3$ is dissolved in an excess of gluconic acid and the CO_2 produced by the dissolution of $CaCO_3$ leaves the hydrogel without causing CO_2 bubbles, which is most likely due to the small size of the $CaCO_3$ particles.

The amount of GDL added to the final mixture was kept at 3.56 times the amount of $CaCO_3$ for a complete dissolution of $CaCO_3$ based on the following reaction:

$$2C_6H_{10}O_6 + CaCO_3 + H_2O \rightarrow C_{12}H_{22}CaO_{14} + CO_2$$

To complete the reaction, 2 mol of GDL is needed to dissolve 1 mol of $CaCO_3$. The molar mass of GDL and $CaCO_3$ is 178 g/mol and 100 g/mol, respectively. Thus, if 1 g of $CaCO_3$ is taken, 3.56 g of GDL is needed to complete the reaction.

The curing time of the materials was around 10 to 20 minutes depending on the concentration of the $CaCO_3$ and GDL used. For example, when using 0.2 wt% $CaCO_3$ and 0.71 wt% GDL, the curing time was around 15 minutes. When increasing or reducing the concentration of $CaCO_3$ and GDL, it took a shorter or longer time to cure.

Samples for compression tests

Compression tests were performed to evaluate the mechanical properties of different material variations. Cylindrical samples with a diameter and height of 10 mm were prepared by casting as shown in Figure S2. The material used for each sample is around 0.79 mL. Before the measurement, the diameter and height of the sample were remeasured to reduce the errors. The compression speed was set at 10 mm/min. The force-displacement curve was recorded from the test machine and converted to a stress-strain curve according to the following equations:

$$\sigma = \frac{F}{A}$$

$$\varepsilon = \frac{\Delta L}{L_0}$$

Where σ is the stress, F is the force, A is the basal area of the sample, ε is the strain, ΔL is the displacement, L_0 is the height of the sample. The elastic modulus was determined using Hooke's law within the elastic range between 1% to 10% strain.

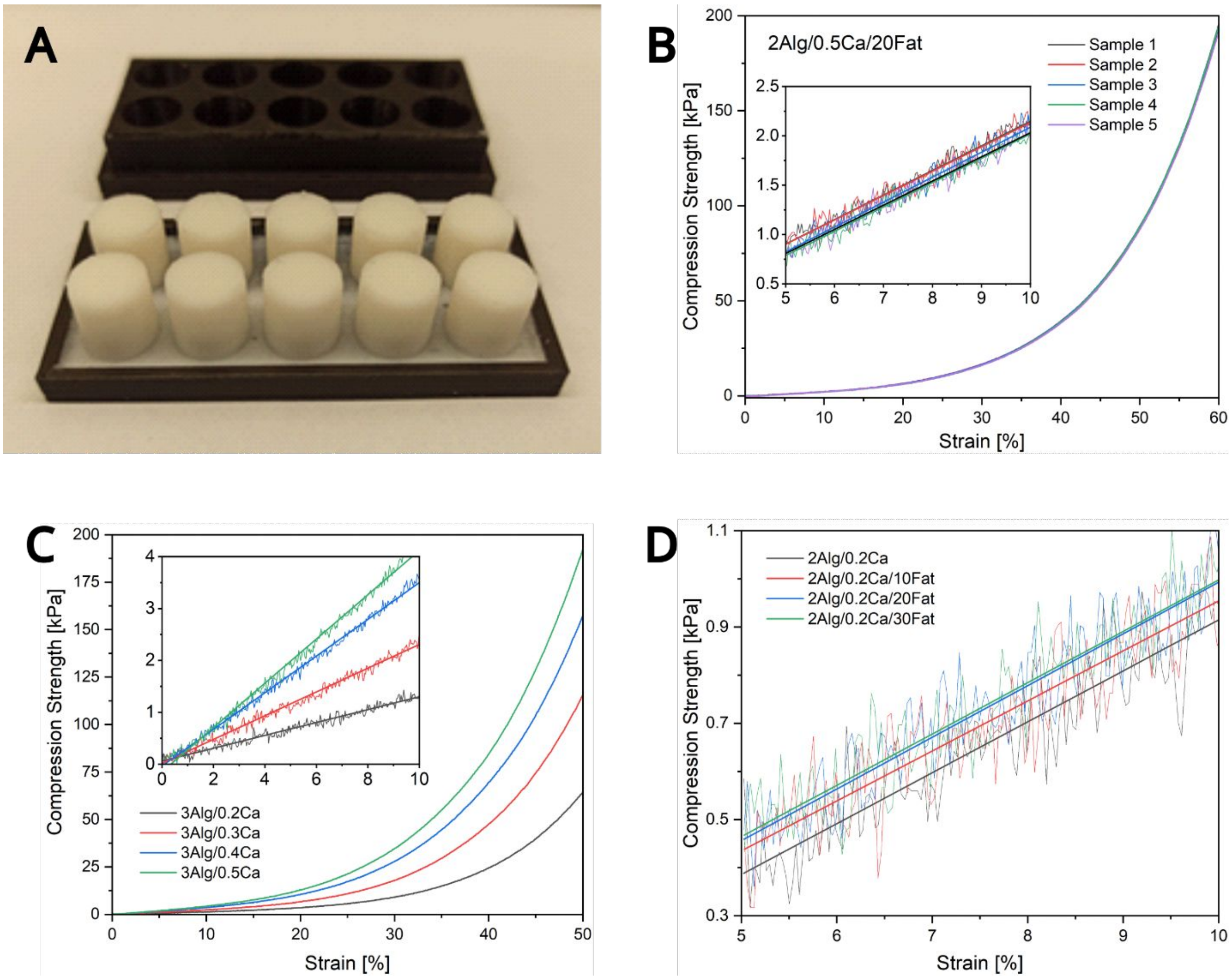

Figure S2: (A) Cylindrical samples with a diameter and height of 10 mm. (B) Compression curves of material variation: 2 wt% alginate / 0.5 wt% Ca / 20 wt% fat. (C) Compression curves of material variations: 3 wt% alginate with different crosslinking degrees (0.2 wt%, 0.3 wt%, 0.4 wt%, and 0.5 wt% $CaCO_3$). (D) Compression curves of material variations: 2 wt% alginate with the same crosslinking degrees and different fat addition (0 wt%, 10 wt%, 20 wt%, and 30 wt% fat).

Process of the data obtained from computed tomography

In order to evaluate the CT attenuation of the material variations, samples were measured by Symbia Intevo 6 SPECT/CT Hybridscanner (Siemens AG, Munich, Germany). The acquired image data sets were archived in DICOM format. The Hounsfield unit was calculated from the DICOM files by self-developed Python-based software. The user interface of the software is shown in Figure S3 which demonstrates the processing of the torso phantom. By clicking on the sagittal, transversal, and frontal images, the center of the calculating area is defined. By defining the sphere radius, the mean Hounsfield unit and the standard deviation are automatically calculated within the sphere area. The software codes are available on request from the authors.

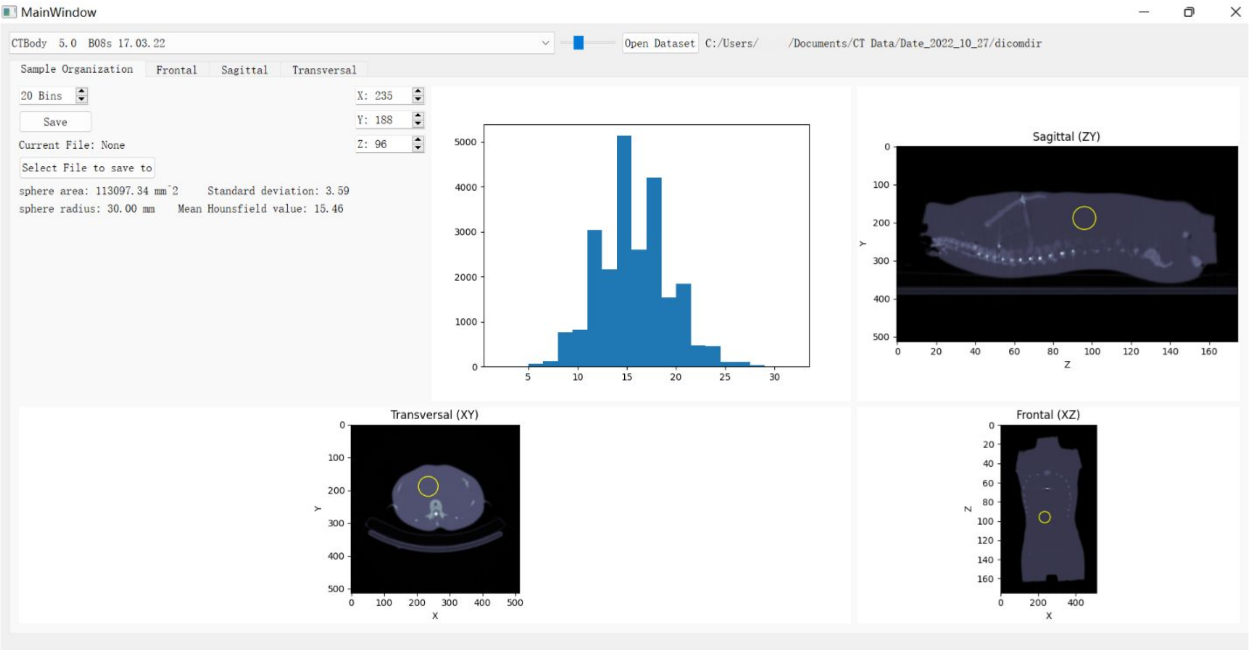

Figure S3: The user interface of the self-developed Python-based software, which shows the Hounsfield unit and the standard deviation of the selected spherical area of the torso phantom.

Torso phantom preparation

Towards the real application, a life-sized torso phantom with a kidney tumor was created by the following steps shown in Figure S4. The torso mold consisting of two pieces (front and back) was created using glass fiber and epoxy with the help of the commercially available acrylic torso shell (Heinrich Woerner GmbH, Leingarten, Germany). The alginate-based blood vessel, kidney, and tumor phantoms were prepared with different amounts of coconut fat addition. 1 wt % red pigment (Fe_2O_3) was added to modify the color. The alginate tumor was embedded into one of the kidneys during preparation. The commercially available polyvinyl chloride skeleton (TecTake GmbH, Igersheim, Germany) was placed into the back piece of the torso mold. Before placing the skeleton into the mold, all the metal rods and screws were replaced with polyvinyl chloride rods and Pattex glue (Henkel AG & Co. KGaA, Dusseldorf, Germany) to reduce the artifacts in the CT measurements. The alginate organs were fixed to the skeleton with polyvinyl chloride cling film. After the fixation of the organs and skeleton, the front piece of the torso mold was put on and closed with clips. The prepared alginate material was poured into the torso mold and kept for 24 h before CT measurement. In total, 40 kg of material was needed to prepare the final torso phantom.

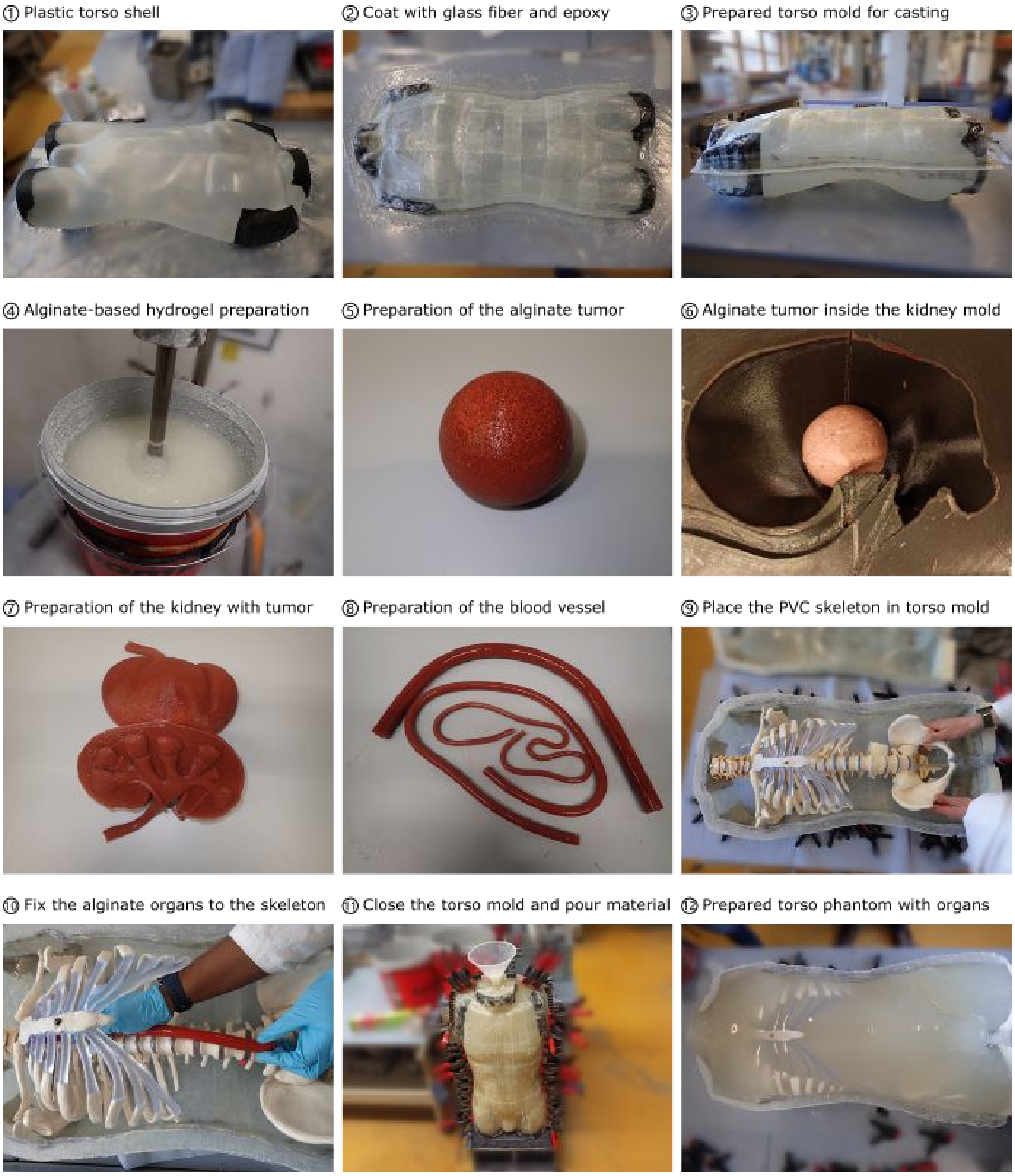

Figure S4: The preparation steps for the torso phantom with organs and a kidney tumor by casting.

CT measurements

The cast samples and the prepared torso phantom were measured under CT as shown in Figure S5. The cuboid shape samples were prepared in a dimension of 10 cm x 12 cm x 7 cm. The material

used for each sample was around 0.84 Liter. The material used for making the torso phantom was around 40 Liters.

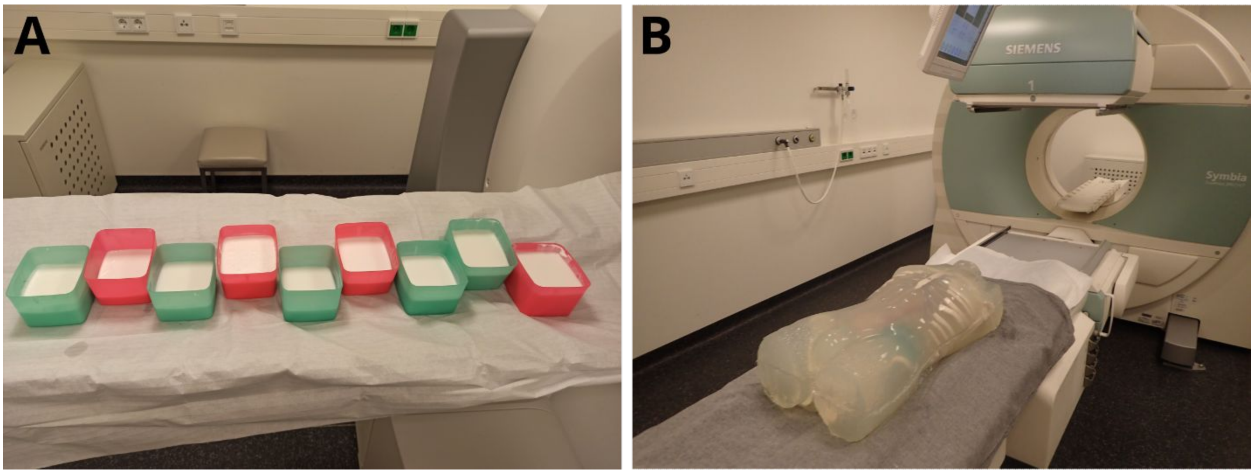

Figure S5: (A) The cast samples and (B) torso phantom under CT measurements.

US measurements

The cast samples with layers of different material variations were measured under US as shown in Figure S6.

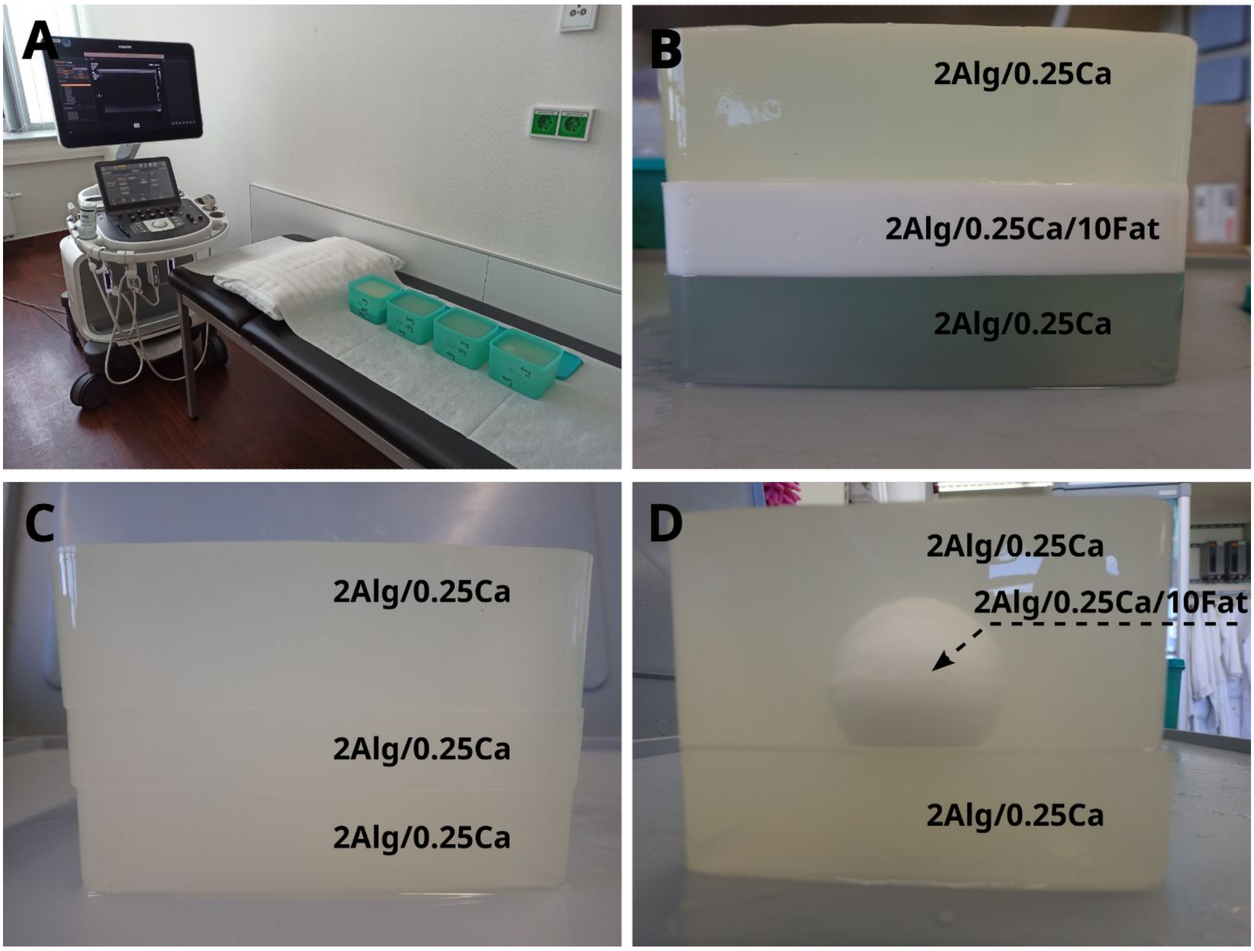

Figure S6: (A) Cast samples under US measurements. (B-D) Cast samples with layers of different material variations for US measurements.

Robotic surgery

The alginate kidney with an alginate tumor was operated under robotic surgery as shown in Figure S7. Torso phantom with a kidney tumor was operated under robotic surgery (Figure S7).

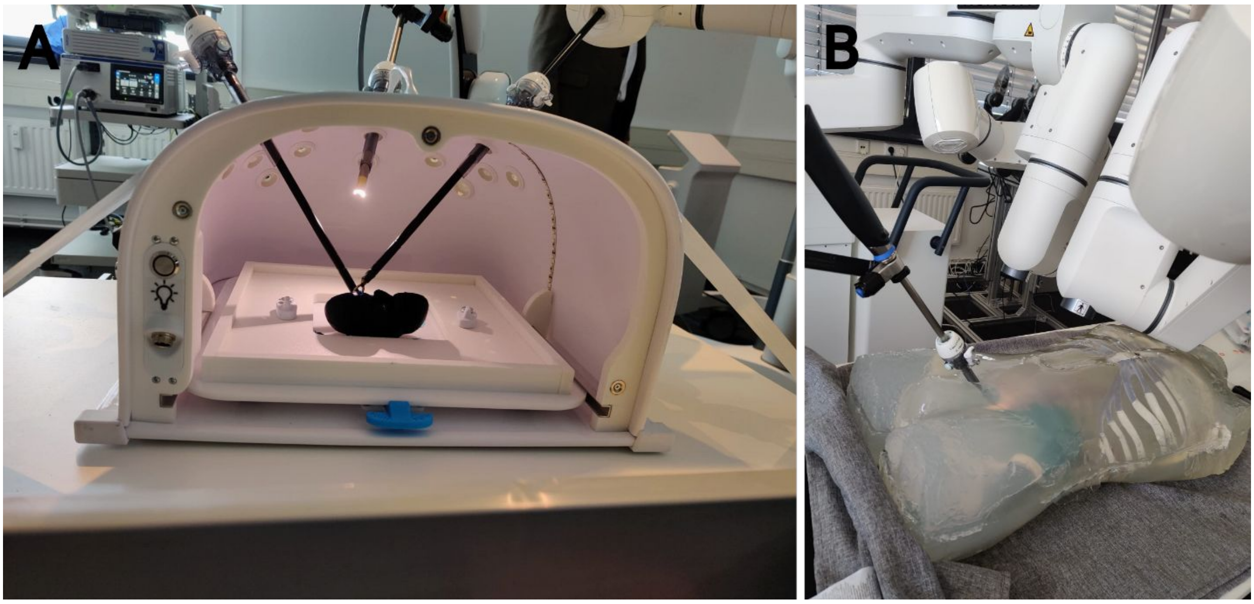

Figure S7: (A) The alginate kidney with a tumor under robotic surgery. (B) Torso phantom with kidney tumor under robotic surgery.

Anti-mold feature of the coconut fat

To investigate the effect of coconut fat on preventing the growth of mold (fungi) on alginate composite, two material variations (2 wt% alginate, 2 wt% alginate/10 wt% coconut fat) were prepared. The samples of each material variation were stored in four different conditions: (1) stored at room temperature, (2) stored in the fridge, (3) coconut fat dip-coated and then stored at room temperature, (4) coconut fat dip-coated and then stored in the fridge. The appearance of samples was recorded every week by camera. Figure S8 shows the pictures of the samples in the first and fifth week, where for both variations the samples dip-coated with coconut fat and stored at room temperature are free of mold even after 5 weeks. Figure S9 shows the sample with coconut fat which is free of mold even after 12 week. The idea for the application would be to create the phantom from patient data, immediately measure US and CT contrast and then the surgeons would have time to practice the surgery. Since the time between the diagnosis of a patient and the surgery is usually around a few days to a few weeks (e.g. 5 weeks for breast cancer[12]), the stability of the material system is sufficient.

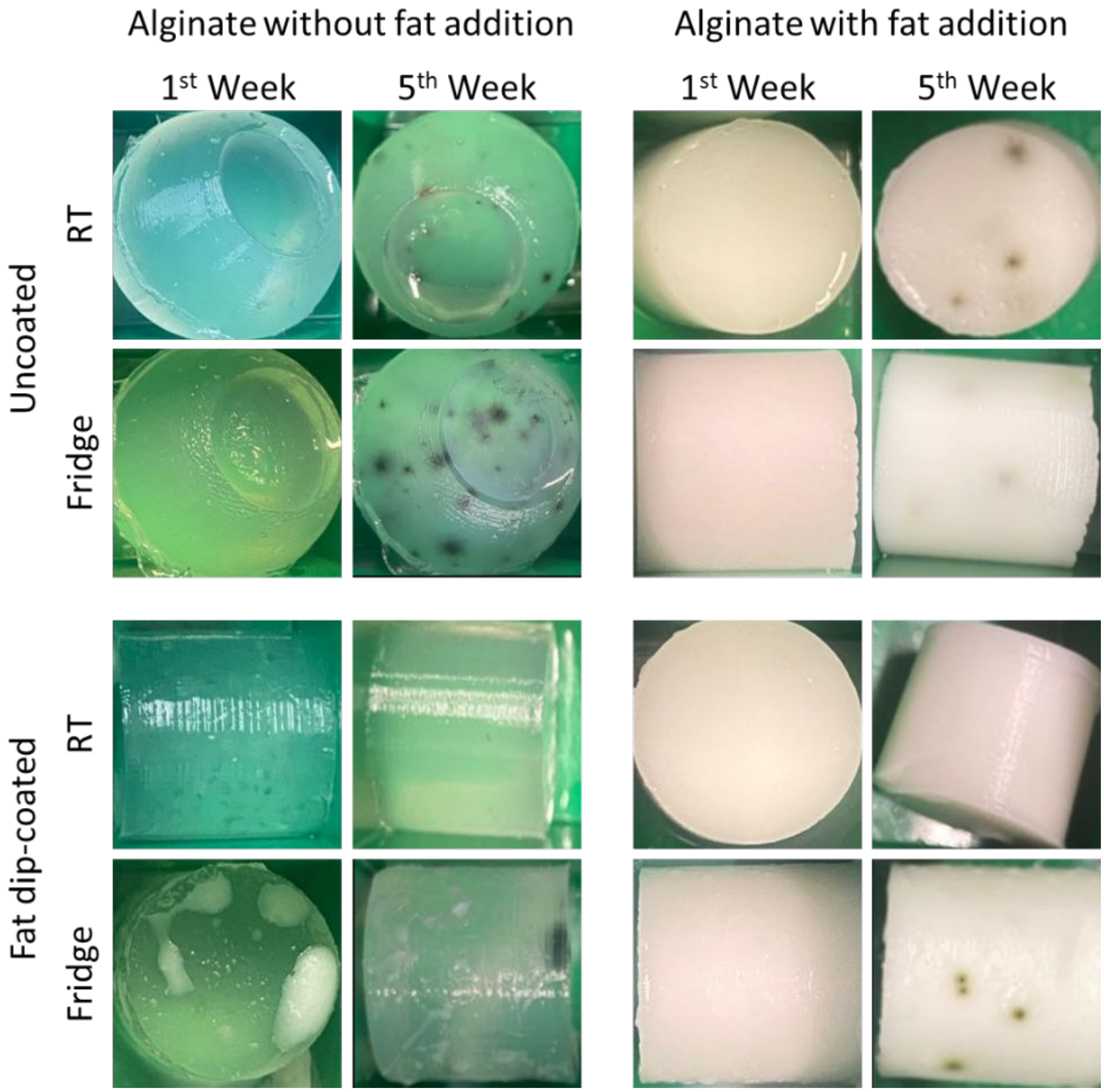

Figure S8: Anti-mold experiment of coconut fat.

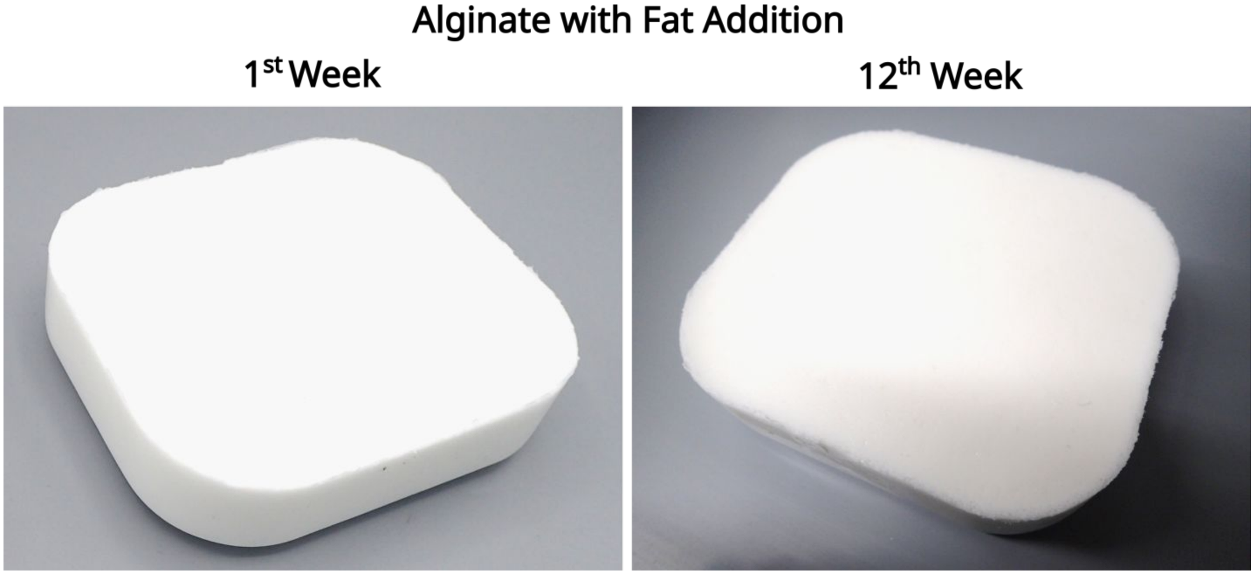

Figure S9: Sample with coconut fat in the 1st and 12th week.

Tunable material properties in wide range

Figure S10 demonstrates that a wide range of elastic modulus, CT attenuation, and shear wave velocity can be achieved by adjusting the degree of crosslinking and the addition of coconut fat. The values obtained from real tissue and organs are represented by dashed lines, and are observed to fall within the range of values attainable by the composite materials.

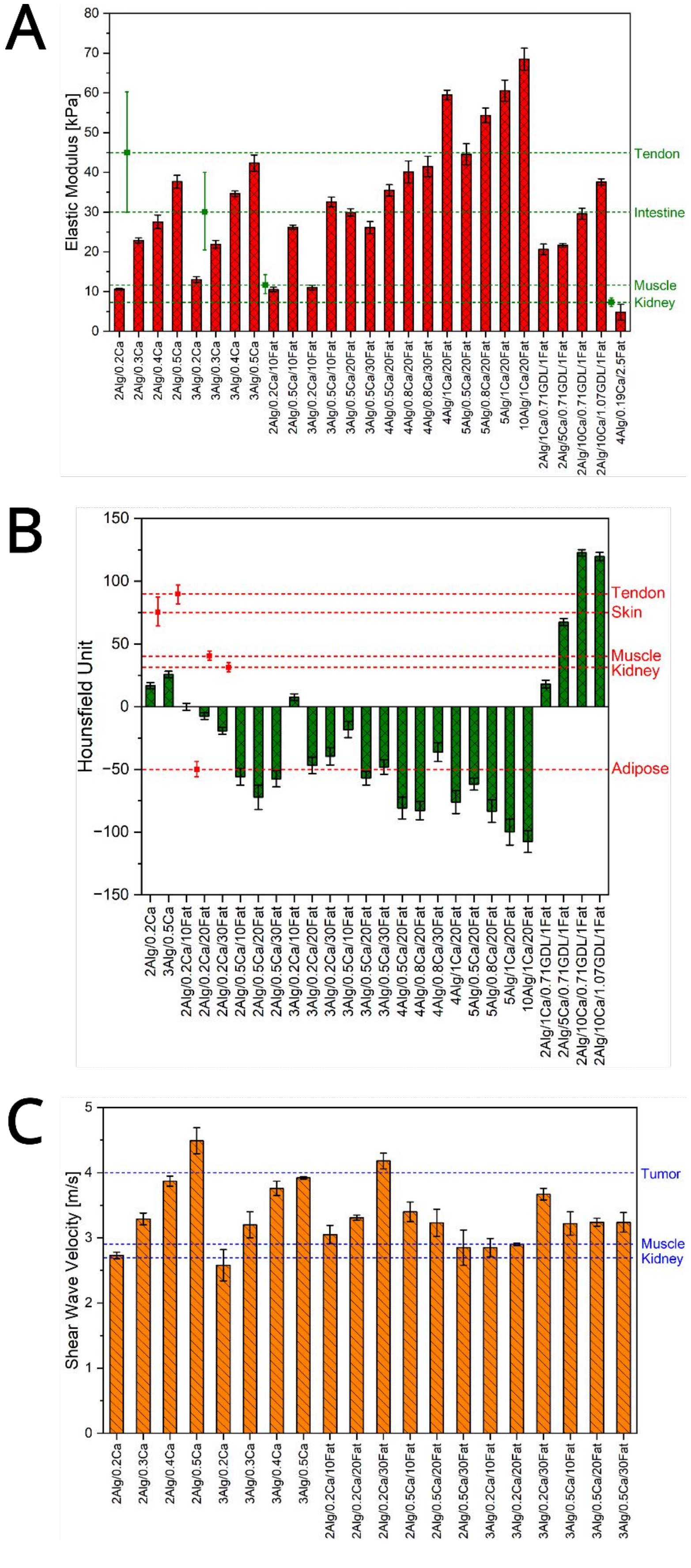

Figure S10: (A) Elastic modulus, (B) CT attenuation, and (C) Shear wave velocity of material variations. The values for the real tissue and organs are obtained from previous work.

Independently tailorable material properties

The composite materials can be cast into different and suitable sizes and shapes for a specific application. As shown in Figure S11A, the calcium ion crosslinked alginate with fat addition was cast into cylindrical and spherical shapes in varied sizes. The mechanical properties of the composite can be tuned by adjusting the crosslinking degree of the polymer network which was achieved by adding different amounts of crosslinker (i.e., Ca^{2+}). Figure S11B shows the compressive curves of material variations with different crosslinking degrees (Ca^{2+} amount), where both the compression strength and elastic modulus increased by increasing the $CaCO_3$ amount from weight percent 0.2 wt% to 0.5 wt%. In order to adjust the CT attenuation of the composite, coconut fat with a lower density (~0.9 g/cm^3) than water (1 g/cm^3) was mixed into the alginate hydrogel. Figure S11C shows CT images of material variations with different amounts of coconut addition. By applying the window level of 40 and window width of 350, it can be clearly seen that the CT contrast was achieved by lowering the density with the addition of coconut fat. Figure S11D shows the US images of samples with different layers of material variations. The contrast can be clearly observed from sonography due to the different shear wave velocities of the US in layers of different crosslinking degrees.

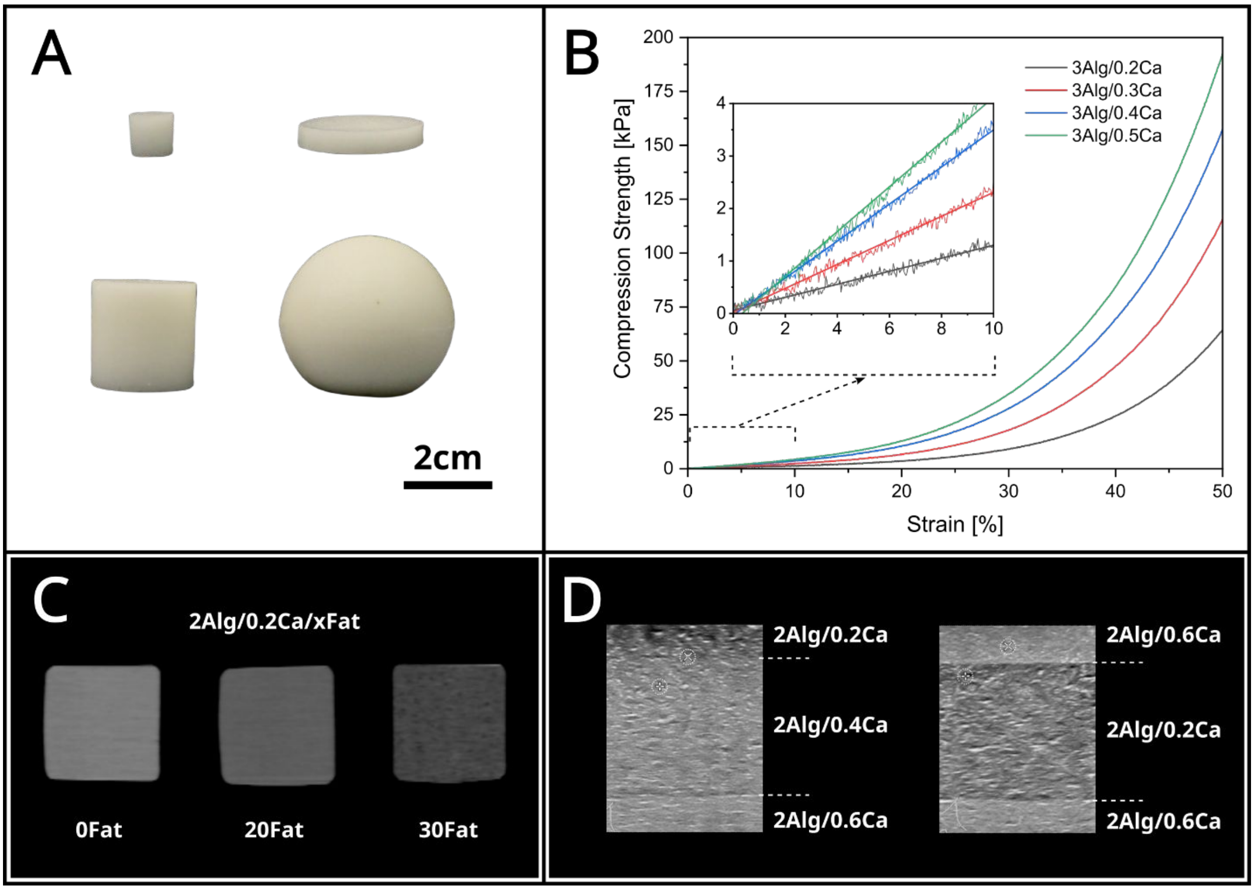

Figure S11: (A) Samples of alginate with fat addition cast in different sizes and shapes. (B) Compression curves of material variations: 3 wt% alginate with different crosslinking degrees (0.2 wt%, 0.3 wt%, 0.4 wt%, and 0.5 wt% $CaCO_3$). (C) CT images of material variations: 2 wt% alginate crosslinked with 0.2 wt% $CaCO_3$ and with different coconut fat addition (0 wt%, 20 wt%, and 30 wt%). The window level and window width are set at 40 and 350, respectively. (D) US images of material variations: 2 wt% alginate with different crosslinking degree (0.2 wt%, 0.4 wt%, and 0.6 wt%) prepared in layers.

Figure S12 was presented as a supplement to Figure 3.

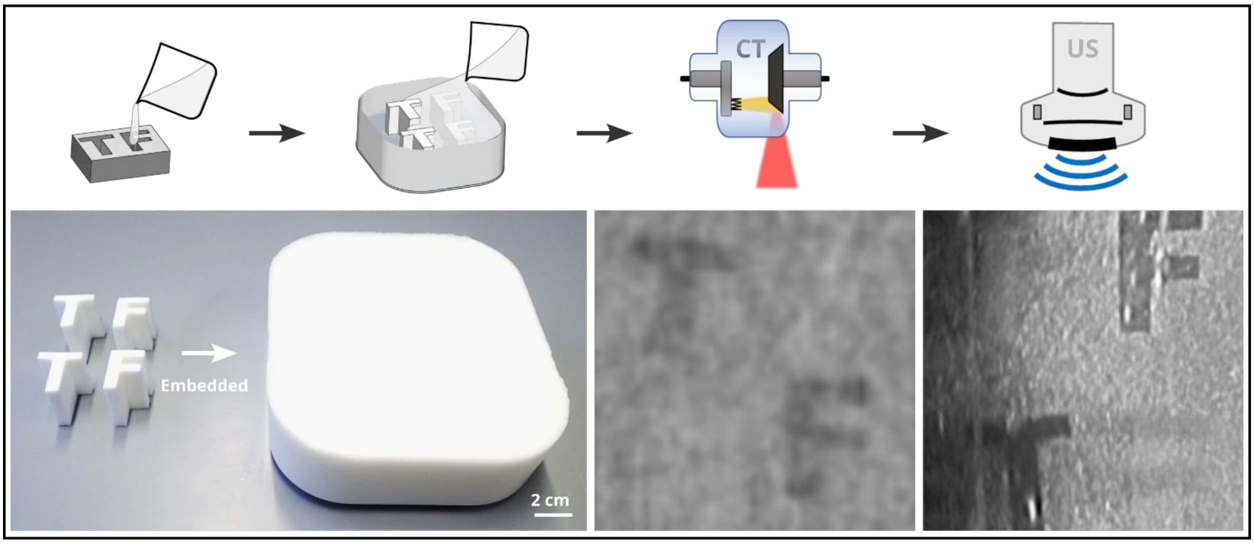

Figure S12: Samples of 2 wt% alginate with different fat addition and crosslinking degrees (10 wt% and 30 wt% fat, 0.25 wt% and 0.75 wt% $CaCO_3$) cast in the shape of letters T and F, embedded in a matrix variation (2 wt% alginate/0.25 wt% $CaCO_3$/10 wt% fat). The CT and US images of the matrix contain the letters T and F. The window level and window width of the CT image are set at 40 and 350, respectively.

Scanning electron microscope (SEM)

The microstructure of crosslinked alginate was characterized by SEM (Zeiss Gemini Ultra55 Plus, Carl Zeiss AG, Oberkochen, Germany) at an acceleration voltage of 5 keV. Before placing the samples into the SEM chamber, they were freeze-dried for 48 h with the freeze dryer (Martin Christ Gefriertrocknungsanlagen GmbH, Osterode am Harz, Germany) and sputtered with a thin gold layer (<20 nm) for conductivity using a sputter coater (Vac Coat Ltd., London, UK).

Figure S13 shows the microstructure of the Ca^{2+} crosslinked alginate, where the interconnected alginate walls divide the bulk material into a large number of closed units in the dimension of hundred micrometers.

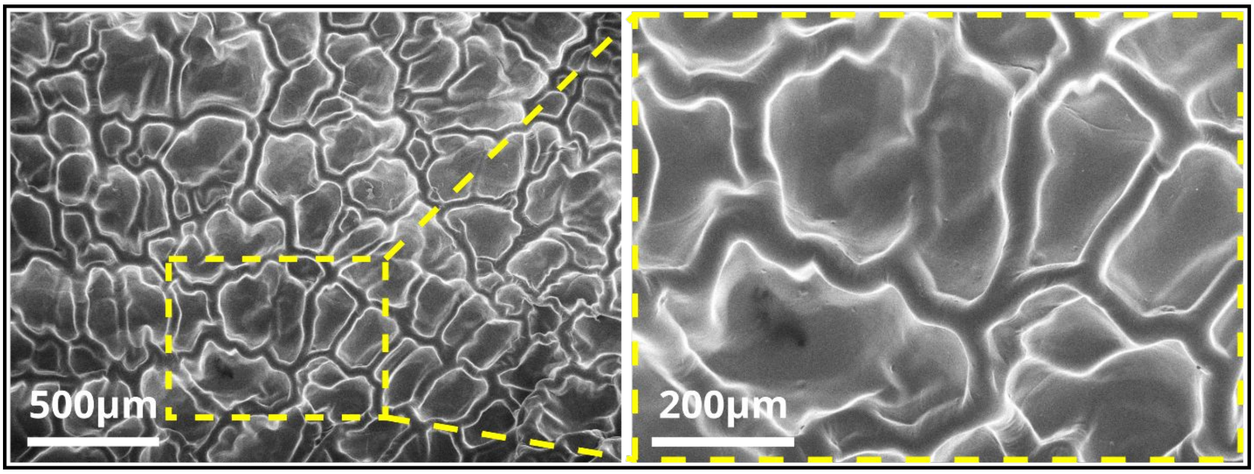

Figure S13: SEM images of sodium alginate crosslinked with Ca^{2+} in different magnifications.

Torso phantom and surgical practice

Figure S14A presents the US image of the tumor within a kidney phantom, and illustrates the contrast caused by the differences in shear wave velocity. Figure S14B illustrates an example application of the developed torso phantom, which demonstrates the tumor resection performed using robotic surgery.

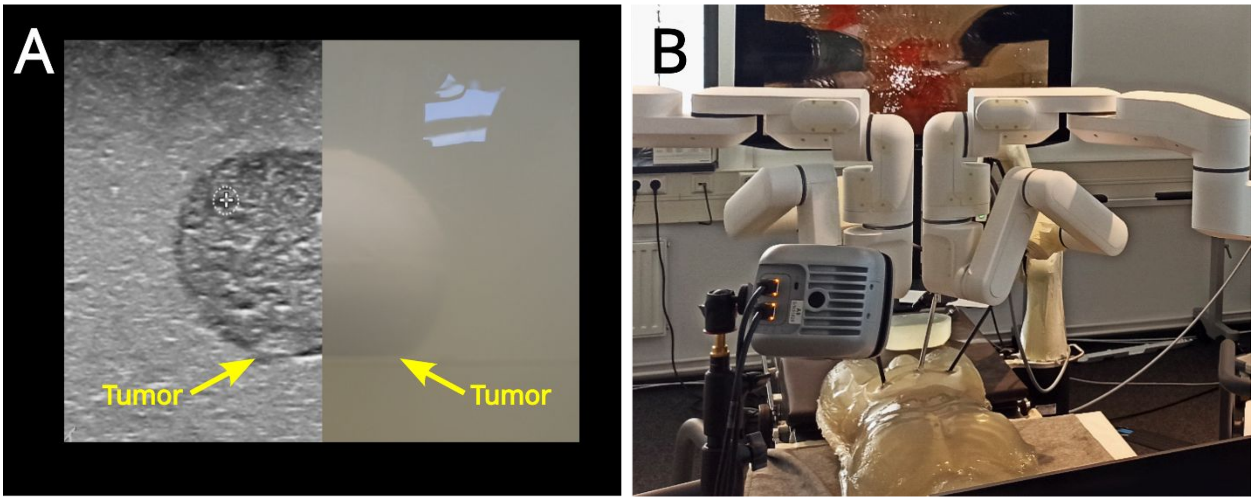

Figure S14: (A) US image (left) and photograph (right) of the phantom with tumor. (B) Tumor resection of the phantom by robotic surgery.

Raman spectroscopy

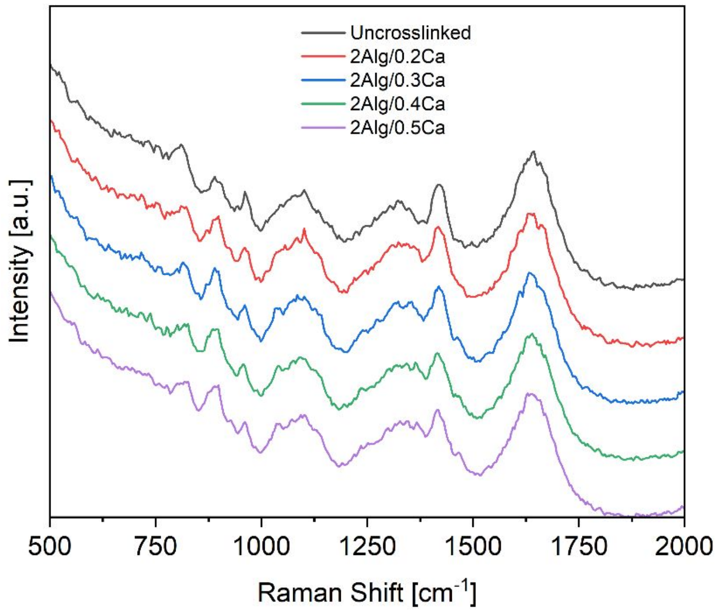

Figure S15: Raman spectra of sodium alginate (uncrosslinked) and alginate crosslinked with different Ca^{2+} concentrations.

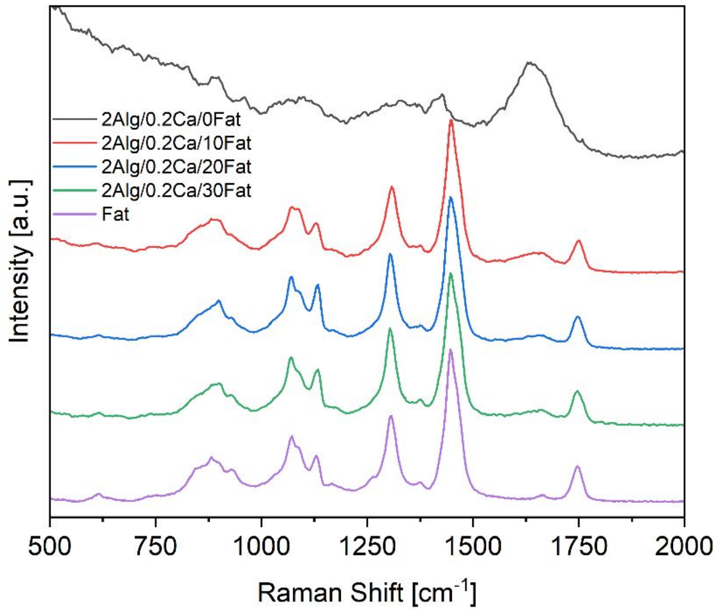

Figure S16: Raman spectra of Ca^{2+} crosslinked alginate, coconut fat and alginate with different fat addition (10 wt%, 20 wt% and 30 wt%).

References

(1) Hungr, N.; Long, J.-A.; Beix, V.; Troccaz, J. A Realistic Deformable Prostate Phantom for Multimodal Imaging and Needle-Insertion Procedures. *Med Phys* **2012**, *39* (4), 2031–2041. https://doi.org/10.1118/1.3692179.

(2) Mackle, E. C.; Shapey, J.; Maneas, E.; Saeed, S. R.; Bradford, R.; Ourselin, S.; Vercauteren, T.; Desjardins, A. E. Patient-Specific Polyvinyl Alcohol Phantom Fabrication with Ultrasound and X-Ray Contrast for Brain Tumor Surgery Planning. *Journal of Visualized Experiments* **2020**, No. 161. https://doi.org/10.3791/61344.

(3) Ruschin, M.; Davidson, S. R. H.; Phounsy, W.; Yoo, T. S.; Chin, L.; Pignol, J.-P.; Ravi, A.; McCann, C. Technical Note: Multipurpose CT, Ultrasound, and MRI Breast Phantom for Use in Radiotherapy and Minimally Invasive Interventions. *Med Phys* **2016**, *43* (5), 2508–2514. https://doi.org/10.1118/1.4947124.

(4) He, Y.; Liu, Y.; Dyer, B. A.; Boone, J. M.; Liu, S.; Chen, T.; Zheng, F.; Zhu, Y.; Sun, Y.; Rong, Y.; Qiu, J. 3D-Printed Breast Phantom for Multi-Purpose and Multi-Modality Imaging. *Quant Imaging Med Surg* **2019**, *9* (1), 63–74. https://doi.org/10.21037/qims.2019.01.05.

(5) Laing, J.; Moore, J.; Vassallo, R.; Bainbridge, D.; Drangova, M.; Peters, T. Patient-Specific Cardiac Phantom for Clinical Training and Preprocedure Surgical Planning. *Journal of Medical Imaging* **2018**, *5* (02), 1. https://doi.org/10.1117/1.jmi.5.2.021222.

(6) Pacioni, A.; Carbone, M.; Freschi, C.; Viglialoro, R.; Ferrari, V.; Ferrari, M. Patient-Specific Ultrasound Liver Phantom: Materials and Fabrication Method. *Int J Comput Assist Radiol Surg* **2015**, *10* (7), 1065–1075. https://doi.org/10.1007/s11548-014-1120-y.

(7) Bhatti, S. S.; Singh, J. 3D Printing of Biomaterials for Biomedical Applications: A Review. *International Journal on Interactive Design and Manufacturing (IJIDeM)* **2023**. https://doi.org/10.1007/s12008-023-01525-z.

(8) McGarry, C. K.; Grattan, L. J.; Ivory, A. M.; Leek, F.; Liney, G. P.; Liu, Y.; Miloro, P.; Rai, R.; Robinson, A.; Shih, A. J.; Zeqiri, B.; Clark, C. H. Tissue Mimicking Materials for Imaging and Therapy Phantoms: A Review. *Phys Med Biol* **2020**. https://doi.org/10.1088/1361-6560/abbd17.

(9) Chiarelli, P.; Lanatà, A.; Carbone, M.; Domenici, C. High Frequency Poroelastic Waves in Hydrogels. *J Acoust Soc Am* **2010**, *127* (3), 1197–1207. https://doi.org/10.1121/1.3293000.

(10) Safety Date Sheet, Polyurethane, Nedform b.v., Geleen, Netherlands.

(11) Safety Data Sheet, Polyurethane, Polytek Development Corp., Pennsylvania, USA.

(12) Chau, I.; Kelleher, M. T.; Cunningham, D.; Norman, A. R.; Wotherspoon, A.; Trott, P.; Rhys-Evans, P.; Rovere, G. Q. Della; Brown, G.; Allen, M.; Waters, J. S.; Haque, S.; Murray, T.; Bishop, L. Rapid Access Multidisciplinary Lymph Node Diagnostic Clinic: Analysis of 550 Patients. *Br J Cancer* **2003**, *88* (3), 354–361. https://doi.org/10.1038/sj.bjc.6600738.
